# Dental Resin Composites Modified with Chitosan: A Systematic Review

**DOI:** 10.3390/md23050199

**Published:** 2025-05-01

**Authors:** Wojciech Dobrzyński, Paweł J. Piszko, Jan Kiryk, Sylwia Kiryk, Mateusz Michalak, Agnieszka Kotela, Julia Kensy, Witold Świenc, Natalia Grychowska, Jacek Matys, Maciej Dobrzyński

**Affiliations:** 1 Department of Dentofacial Orthopedics and Orthodontics, Division of Facial Abnormalities, Wroclaw Medical University, Krakowska 26, 50-425 Wroclaw, Poland; wojt.dobrzynski@wp.pl; 2 Department of Biomedical Engineering, Faculty of Fundamental Problems of Technology, Wrocław University of Science and Technology, Wybrzeże Wyspiańskiego 27, 50-370 Wrocław, Poland; pawel.piszko@pwr.edu.pl; 3 Dental Surgery Department, Wroclaw Medical University, Krakowska 26, 50-425 Wroclaw, Poland; jan.kiryk@umw.edu.pl; 4 Department of Pediatric Dentistry and Preclinical Dentistry, Wroclaw Medical University, Krakowska 26, 50-425 Wroclaw, Poland; maciej.dobrzynski@umw.edu.pl; 5 Medical Center of Innovation, Wroclaw Medical University, Krakowska 26, 50-425 Wroclaw, Poland; mateusz.michalak92@gmail.com (M.M.); kotela.agnieszka@gmail.com (A.K.);; 6 Faculty of Dentistry, Wroclaw Medical University, Krakowska 26, 50-425 Wroclaw, Poland; julia.kensy@student.umw.edu.pl; 7 Department of Dental Prosthetics, Wroclaw Medical University, Krakowska 26, 50-425 Wroclaw, Poland; natalia.grychowska@umw.edu.pl

**Keywords:** antibacterial, chitosan, mechanical properties, resin composite

## Abstract

Objective: This systematic review aims to evaluate the impact of incorporating chitosan into dental resin composites on their mechanical, antibacterial, and physicochemical properties. Methods: A comprehensive search of PubMed, Scopus, and Web of Science databases was conducted in March 2025 using the following keywords: resin, composite, and chitosan. The inclusion criteria comprised in vitro studies in dentistry evaluating chitosan as a composite additive with full-text availability in English. Data extraction and quality assessment were performed independently by multiple reviewers using standardized tools, and study quality was assessed based on predefined criteria. Results: Seventeen studies met the inclusion criteria. Chitosan concentrations in the composites ranged from 0.25 wt% to 20 wt%. Antibacterial activity was confirmed in six studies, especially against *S. mutans*, *S. sanguinis*, and *L. acidophilus*. Mechanical properties such as fracture toughness, hardness, and compressive strength were generally improved at lower concentrations of chitosan. However, increased chitosan levels were associated with decreased flexural strength and increased microleakage. Shear bond strength (SBS) was unaffected by chitosan in low concentrations (up to 0.25%), while higher concentrations reduced SBS. Fluoride release capacity was assessed in one study, with no significant differences observed. Conclusion: Chitosan-modified dental resin composites exhibit promising antibacterial and mechanical enhancements at low concentrations. However, higher chitosan levels may compromise certain mechanical and adhesive properties. These findings suggest a need for standardized methodologies and further research on long-term clinical implications and fluoride release.

## 1. Introduction

Composite resins are widely used in restorative dentistry due to their ability to closely mimic the appearance and function of natural teeth [1,2]. These materials consist of an organic polymer matrix, typically composed of bisphenol A-glycidyl methacrylate (Bis-GMA), urethane dimethacrylate (UDMA), or triethylene glycol dimethacrylate (TEGDMA), combined with inorganic fillers such as silica, quartz, or zirconia to enhance mechanical properties and wear resistance [2]. The filler content and particle size influence key properties, including strength, polishability, and translucency, making composite resins suitable for various clinical applications [3,4]. They are commonly used for direct restorations in both anterior and posterior teeth, offering esthetic and conservative treatment options. Additionally, composite resins are employed in procedures such as veneers, diastema closures, core build-ups, and the cementation of indirect restorations, providing versatility in dental practice [5]. Their ability to bond to tooth structures via adhesive systems enables minimally invasive restorations, preserving sound enamel and dentin while ensuring optimal retention [6,7]. Over the years, advancements in composite resin formulations have led to improvements in durability, color stability, and wear resistance, further expanding their applications in both restorative and cosmetic dentistry [8]. Despite these benefits, conventional composites still face challenges such as polymerization shrinkage and bacterial adhesion, which may compromise the longevity of restorations [9,10]. Therefore, ongoing research focuses on modifying composite resins with bioactive and antimicrobial agents to enhance their clinical performance [11].

**Figure 1:**
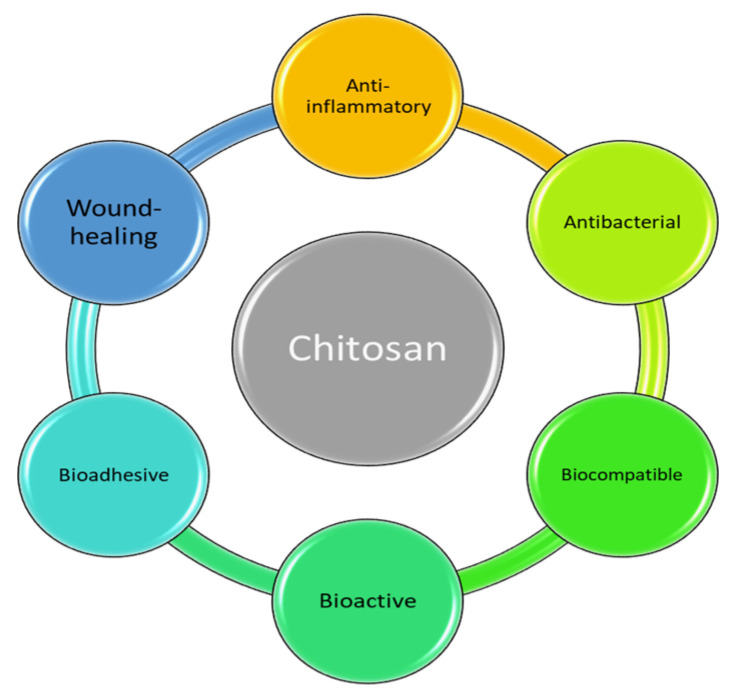
Advantages of chitosan.

Chitosan (C/CH/CS), a biopolymer derived from the deacetylation of chitin, has gained significant attention in dentistry due to its antimicrobial, biocompatible, and bioactive properties [12,13]. Its cationic nature enables interaction with negatively charged bacterial cell membranes, leading to cell disruption and the prevention of biofilm formation—an essential factor in reducing secondary caries and periodontal infections [14]. Additionally, chitosan exhibits anti-inflammatory and wound-healing capabilities by promoting fibroblast proliferation and modulating inflammatory responses, making it beneficial for tissue regeneration [13,15]. It also contributes to remineralization by binding to calcium and phosphate ions, thereby aiding in the repair of demineralized dental tissues [16,17]. When incorporated into dental composite resins, chitosan enhances mechanical properties such as flexural and compressive strength while maintaining esthetic qualities [18]. Furthermore, its bioadhesive properties improve the interaction between resin and dentin, potentially increasing the longevity of restorations [19]. Owing to these advantages, researchers are increasingly exploring the integration of chitosan into dental materials to develop next-generation composites with improved antibacterial and mechanical performance [20] (see Figure 1).

Dental resin composites have undergone substantial evolution since their introduction, with their composition continuously modified to enhance mechanical properties, biocompatibility, and clinical longevity [21,22,23,24,25]. The incorporation of various additives into resin matrices has led to significant improvements, including the use of antimicrobial agents (e.g., chlorhexidine, silver nanoparticles, zinc oxide nanoparticles) [26,27,28,29,30], bioactive fillers (e.g., calcium phosphates, amorphous calcium phosphate (ACP), and bioactive glass particles) [27,31,32], reinforcing fibers (e.g., glass fibers, carbon fibers, zirconia fibers) [33,34], and nanoparticles (e.g., silica, titanium dioxide, zirconium oxide) [28,35,36,37]. These modifications have broadened the clinical applications of resin composites while maintaining both durability and biocompatibility in the oral environment. Chitosan, a natural polysaccharide that is derived from chitin, has become a subject of significant interest in the field of dentistry due to its potential application as an additive in dental resin composites. This interest can be attributed to the following properties: inherent antimicrobial activity, biocompatibility, and biodegradability. Its molecular weight influences its reactivity and activity with other composite components [38]. Moreover, processing methods—such as dispersion techniques and chemical modifications—are essential for achieving a homogeneous distribution of chitosan within the resin matrix [18,39,40]. Environmental factors, including pH and temperature, also influence the stability and effectiveness of chitosan-modified composites [13,41,42].

Despite the growing body of research exploring various resin additives—including chitosan—in dental composites, a notable gap remains in the literature regarding a comprehensive systematic review that synthesizes the current state of knowledge on this topic. This review aims to critically evaluate the available evidence on chitosan-modified dental resin composites, with a focus on assessing the impact of chitosan incorporation on the material’s physical, mechanical, and antibacterial properties. While numerous individual studies have examined specific aspects of chitosan integration [13,19,38,39,40,41,42,43], a holistic analysis encompassing the wide range of variables influencing these outcomes is lacking. Therefore, this systematic review aims to critically evaluate the current evidence regarding the incorporation of chitosan into dental resin composites, focusing on its influence on mechanical and antibacterial properties. The novelty of this review lies in its comprehensive comparison of different forms, concentrations, and preparation techniques of chitosan-modified composites. Our findings forecast that low concentrations of chitosan can enhance antibacterial efficacy without compromising mechanical performance, while higher concentrations may adversely affect key properties. These insights provide a scientific foundation for optimizing the use of chitosan in next-generation dental composites and guide future research directions.

## 2. Results

### 2.1. Study Selection

An initial database search yielded 557 scientific articles that potentially met the inclusion criteria for the present systematic review. Subsequent to the elimination of duplicates, the number of entries was reduced to 304. The subsequent screening of titles and abstracts resulted in the exclusion of 282 entries, with 35 of these being excluded on the basis of the addition of chitosan to different dental materials, 29 due to the nature of the conference report, 19 due to the review article type, 2 due to the fact that they were written in a language other than English, and 197 due to their relevance to fields other than dentistry. Consequently, a total of 22 articles were subjected to a thorough assessment to ascertain their eligibility. Following a thorough examination of the full texts, five articles were rejected due to failure to meet the inclusion criteria (one article was deemed to be related to a field other than dentistry, three articles were rejected due to the addition of chitosan to different dental materials, and one article was rejected due to the lack of access to the full text). Consequently, a total of 17 articles were deemed to have met the necessary criteria and were thus selected for inclusion in the systematic review [18,19,40,44,45,46,47,48,49,50,51,52,53,54,55,56,57]. The scope of these articles pertained to dental resin composites modified with chitosan.

### 2.2. General Characteristics of the Included Studies

The primary objective of this systematic review was to evaluate the implications of adding chitosan to dental resins on their properties and potential clinical applicability. The included studies varied in terms of the form and concentration of chitosan used, as well as in the assessed material properties. General study characteristics are presented in Table 1, while detailed evaluations, including chitosan concentration, antibacterial activity, mechanical performance, bond strength, and fluoride release, are summarized in Table 2. The concentration of chitosan incorporated into dental resins ranged from 0.25 wt% to 20 wt%.

#### 2.2.1. Antibacterial Activity

Antibacterial activity was assessed in nine articles. Chitosan-containing resins inhibited the growth of *S. mutans* [19,40,44,46,48], *S. sanguinis* [48], and *L. acidophilus* [48]. No antibacterial effects were observed against *A. viscosus* [18] and *L. casei* [45]. Notably, Kikuchi et al. [47] reported that crosslinking chitosan with glutaraldehyde significantly improved antibacterial efficacy, highlighting the role of material processing. These findings confirm that chitosan incorporation can enhance the antibacterial performance of dental resins, although the effects are strain-dependent.

#### 2.2.2. Shear Bond Strength

Shear bond strength (SBS) was evaluated in five studies [19,48,54,56,57]. Most studies indicated no statistically significant differences between control groups and composites modified with chitosan at concentrations up to 1 wt% [19,48,54,56]. However, Farzanegan et al. [57] reported that the addition of 1.5 wt% chitosan nanoparticles significantly decreased bond strength. Fadhila et al. [19] also demonstrated that an increased addition of cellulose acetate lowered SBS values. These results suggest that low levels of chitosan incorporation preserve adhesive properties, while higher loadings may compromise bond strength.

#### 2.2.3. Hardness

Hardness was evaluated in five studies [18,19,46,50,54]. Ali et al. [18] demonstrated that the addition of 1 wt% chitosan increased microhardness, while 0.5 wt% enhanced the hardness of flowable resins. Kim and Shin [46] observed no differences in hardness depending on the molecular weight of chitosan after water storage. In contrast, Kikuchi et al. [19] and Harmaji et al. [50] reported a decrease in hardness with higher concentrations (≥4 wt%), and Stenhagen et al. [54] observed decreased hardness at 10 wt% and 20 wt% chitosan. These results suggest that while low concentrations of chitosan may preserve or slightly enhance hardness, excessive incorporation can negatively affect surface strength.

#### 2.2.4. Strength and Toughness Properties

Mechanical properties related to strength and toughness, including impact strength, tensile strength, compressive strength, flexural strength, flexural modulus, and fracture toughness, were reported across several studies.

Masoumi et al. [49] found that chitosan-containing composites exhibited greater impact strength compared to controls, with the best performance at 5 wt% chitosan. Lai et al. [44] demonstrated that 2 wt% chitosan/fluoride microparticles significantly improved tensile strength, while Fadhila et al. [19] showed that a combination of 5 wt% cellulose acetate and 7 wt% chitosan maintained tensile properties comparable to controls. Compressive strength remained unaffected by chitosan addition, as reported by Fadhila et al. [19].

Flexural strength findings varied: moderate chitosan concentrations (2–5 wt%) generally maintained or improved flexural strength [40,44,49], whereas higher concentrations led to reductions [54,55]. Long-term hydration improved flexural strength in the majority of materials [40], except in groups containing 0.5 wt% chitosan. Flexural modulus showed similar trends, remaining stable at low concentrations but decreasing with higher chitosan content [40,46,47,55]. Fracture toughness was comparable across groups according to Tanaka et al. [40]; however, Chander and Venkatraman [49] reported increased toughness with 5 wt% chitosan addition. These results suggest that low to moderate chitosan levels can enhance mechanical resilience, but high concentrations may compromise material performance.

#### 2.2.5. Additional Material Properties

Several studies evaluated additional material characteristics, including microleakage, adhesive remnant index (ARI), and fluoride release.

Microleakage was analyzed in two studies [19,52]. Fadhila et al. [19] observed a statistically significant increase in microleakage for chitosan-modified resins, indicating potential deterioration in marginal sealing. In contrast, Deb et al. [52] reported no significant difference compared to controls, suggesting that the impact of chitosan on microleakage may be formulation-dependent. The adhesive remnant index (ARI) was evaluated by Farzanegan et al. [57]. No significant differences were observed between control and chitosan-modified groups, indicating that the failure mode of orthodontic adhesives remains unaffected by the addition of chitosan. Fluoride release was assessed in one study by Lai et al. [44], who found no statistically significant difference between chitosan-containing and unmodified composites. Thus, fluoride release behavior appears to be preserved with the incorporation of chitosan.

The results of this review align well with existing literature on the integration of chitosan in dental materials. Husain et al. [58] emphasized chitosan’s versatility as a dental biomaterial, noting its biocompatibility, biodegradability, and broad-spectrum antimicrobial properties, which support its use in restorative applications. They highlighted its potential to improve mechanical strength and reduce microbial adhesion, particularly when used in low to moderate concentrations—findings consistent with the current review. Similarly, Cicciù et al. [59] demonstrated chitosan’s contribution to enhanced wound healing, antibacterial performance, and biofilm inhibition when incorporated into various dental cements, further supporting the antibacterial outcomes reported here, especially against *S. mutans* and *S. sanguinis*.

Ali et al. [18] observed that adding up to 1% chitosan nanoparticles to resin composites increased hardness, though they found no antibacterial effect against *A. viscosus*, which mirrors the strain-specific results presented in this review. Moreover, Kim et al. [46] investigated the influence of chitosan molecular weight on mechanical properties and observed that composites with low and high molecular weight chitosan had minimal impact on flexural strength, corroborating our finding that the mechanical performance of chitosan-containing resins varies depending on formulation and testing conditions. These comparisons underscore the importance of optimizing chitosan content and type to balance antibacterial effectiveness with desirable mechanical properties. Collectively, these external studies reinforce the current review’s conclusions that chitosan’s performance in dental composites is multifactorial and context-dependent.

### 2.3. Main Study Outcomes

The resin matrix used in the presented studies varied across the articles. Commercial resins utilized in the studies included the following: ClinPro fissure sealant [44], Microhybrid resin Filtek Z350 XT [18,45], Flowable Filtek Z350 resin [18,45], Flow (Denstplysirona) [48], Dental Products of India (product no: 11811) [49], Neo spectra ST [51], Tetric N-Ceram [51], Brilliant NG [52,53], NeoSpectra [56], Transbond XT [57]. The authors’ formulation of resin was also denoted in seven studies [19,40,46,47,50,54,55].

Furthermore, the chitosan was also added in different forms: chitosan/fluoride microparticles [44], commercial chitosan [18,45,46,49,50,51,52,53], chitosan particles [40,48,56,57], chitosan particles loaded with dibasic calcium phosphate (DCPA) [40,47], chitosan extracted from shrimp shells [19], methacrylated chitosan [54], and phosphorylated chitosan/amorphous calcium phosphate nanocomplex [55].

The hydrolytic stability of CS containing composites was reported by Ali et al. as acceptable [45]. Furthermore, during 3 months of incubation in artificial saliva, Deb et al. [52] showcased superior stability of CS containing resin to the control group as no microleakage occurred. Therefore, in light of the above, CS-containing resin composites can be regarded as stable. Nevertheless, none of the evaluated articles tackled the issue of selective leaching of chitosan from the matrix over time.

A direct comparison of the results obtained in the analyzed articles cannot be made since the physico-chemical properties of the matrix and form of chitosan were not uniform. However, general observations extracted in Section 2.2 lead to the following conclusions:

- Antimicrobial activity of chitosan incorporated in dental resins against *S. mutans*
- Addition of chitosan to dental resins either does not affect or lowers the shear bond strength
- The hardness of the material either does not change or lowers with the addition of chitosan
- The flexural modulus of chitosan-containing dental resins tends to follow a trend observed for flexural strength

### 2.4. Quality Assessment

Twelve articles were classified as having a moderate risk of bias [18,19,40,44,45,46,47,49,53,54,55,57], four articles were characterized as having a high risk of bias [50,51,52,56], and only one article was assessed as having a low risk of bias [48] (Table 3).

## 3. Discussion

The aim of this study was to comprehensively evaluate the effect of chitosan incorporation into composite resins on their properties and potential clinical utility. This systematic review analyzed the results of 17 scientific articles. The antibacterial effect of chitosan was assessed in nine studies [18,19,40,44,45,46,47,48,54], while eight studies did not investigate the impact of chitosan-containing resins on biofilm formation [49,50,51,52,53,55,56,57]. The influence of chitosan on mechanical properties was also widely examined. Specifically, shear bond strength (SBS) was evaluated in five studies [19,48,54,56,57], while twelve studies did not assess this parameter [18,40,44,45,46,47,49,50,51,52,53,55]. Among the studies that investigated antibacterial effects, six reported the effect of inhibition of bacterial growth by chitosan-modified resins [19,40,44,46,47,48]. Regarding adhesion, four studies found that the addition of chitosan had no statistically significant effect on SBS [19,48,54,56]. Only one study [44] examined the effect of chitosan on fluoride release, reporting no significant difference between modified and unmodified resins. Overall, this systematic review concluded that chitosan incorporation affects several mechanical properties other than SBS, including microhardness, microleakage, shrinkage, compressive strength, fracture toughness, tensile strength, flexural strength, solubility, and water absorption.

Of the seventeen studies reviewed, nine [18,19,40,44,45,46,47,48,54] evaluated the effect of chitosan incorporation on the antibacterial properties of resin composites. Eight studies did not report the effect of chitosan-containing resins on the development of bacterial biofilm [49,50,51,52,53,55,56,57]. Interestingly, antibacterial activity was observed against several bacterial strains, including S. mutans, S. sanguinis, and L. acidophilus, which are key factors in the formation of cariogenic biofilm in the oral cavity [60]. This antibacterial feature is of significant clinical importance, as it increases the anticariogenic potential of restorative materials, complementing the protective role of fluoride. The antibacterial efficacy of chitosan depends on its form and integration into the resin matrix. For example, Kikuchi et al. [47] crosslinked chitosan with glutaraldehyde. Only in this crosslinked form did the material demonstrate significant antibacterial activity, underlining the importance of processing techniques in optimizing the performance of chitosan-containing composites. Of the nine studies investigating the antibacterial activity of chitosan composites, six showed significant inhibition of bacterial biofilm growth [19,40,44,46,47,48]. For example, Masoumi et al. [48] found that the control group (without chitosan) had 13,800 CFU of S. mutans, while the experimental group had 5,971. Four studies [40,44,46,47] showed that the addition of chitosan at appropriate concentrations had no effect on the important properties of the resins used in dentistry, such as microhardness, cytotoxicity, water sorption, degree of conversion, microleakage, and shrinkage. Furthermore, in addition to demonstrating better antibacterial activity, the addition of chitosan in the study [44] improved the mechanical properties of the material, such as its microhardness and tensile strength. However, in one study [19], a deterioration in mechanical parameters was observed, which was associated with a higher concentration of added chitosan. While the addition of chitosan worsened the above-mentioned parameters in other studies, the differences were not statistically significant. The addition of chitosan offers great potential to improve the properties of filling materials, especially antibacterial properties, without significantly affecting the resin components, thus avoiding the deterioration of other material parameters [48,60,61,62,63,64]. The above studies confirm that it is possible to create a resin with a strong antibacterial effect that meets the requirements for other parameters.

All seventeen of the studies included in the review assessed the effect of adding chitosan on the mechanical properties of dental resin composites. The properties assessed in addition to shear bond strength included microhardness, depth of cure, flexural strength, tensile strength, microleakage, polymerisation shrinkage, compressive strength, water absorption, solubility, impact strength, fracture toughness, and ARI. These properties are highly relevant to the intended use. The results indicated that chitosan incorporation can improve or adversely affect clinically relevant mechanical parameters. For instance, fracture toughness remained unaffected in all studies, though it increased in one study, suggesting the potential to enhance restoration durability under occlusal stress. Resin hardness increased in four studies [18,44,50,55] and decreased in two [19,54], with results largely dependent on chitosan concentration (lower concentrations generally being more favorable). Furthermore, the impact strength of the material increased with the addition of 5% chitosan but not with higher concentrations [49], which is an important improvement for composite fillings that are constantly subjected to crushing forces during chewing. Positive changes in tensile strength occurred, which were also dependent on the chitosan concentration. Adding 2% chitosan improved this parameter. However, a significant drawback was the increase in microleakage observed in one study [19], though another study [52] did not show a statistically significant difference. Given the importance of the long-term retention of the restoration in the tooth, this feature is crucial because greater leakage at the tooth-composite interface increases the risk of secondary caries. Furthermore, flexural strength decreased in three out of four studies evaluating this property [46,54,55], which could negatively impact the material’s performance in stressed areas. Overall, these results suggest that, while chitosan can benefit composite resins, especially at lower concentrations, its effects are not universally positive. The Optimization of formulation parameters is necessary to balance antibacterial benefits with mechanical performance [48].

Out of the seventeen studies reviewed, five investigated the effect of chitosan addition on shear bond strength (SBS). The studies by Fadhila [19], Masoumi [48], Stenhagen [54], and Halkai [56] examined SBS in cases where chitosan was incorporated into both the composite material and the bonding agent. In contrast, the study by Farzanegan [57] evaluated the effect of chitosan added solely to the bonding agent. The findings of Fadhila [19] and Stenhagen [54] indicated no statistically significant difference in SBS between chitosan-modified and unmodified materials. However, studies by Halkai [56] and Farzanegan [57] demonstrated that increasing chitosan concentration in either the bonding agent or the composite led to a decrease in bond strength. Notably, at a low concentration of 0.25%, the addition of chitosan did not negatively affect the push-out bond strength in class II restorations. In fact, such modification provided the composite with enhanced microbiological and mechanical properties, potentially extending the clinical lifespan of restorations. The study by Masoumi [48] further compared the impact of chitosan incorporation into the composite versus the bonding agent. The results showed a slight, non-significant increase in SBS when chitosan was added to the bonding agent, and a slight decrease when added to the composite. This effect may be attributed to the formation of larger interparticle spaces within the composite matrix upon chitosan addition, potentially hindering the establishment of strong adhesive bonds.

There were very few studies that investigated the effect of fluoride release from chitosan-modified resin composites, despite this being an important feature of dental restorative materials. This aspect was evaluated only in the study by Lai et al. [44], in which ion chromatography was used to compare fluoride release between chitosan-modified and unmodified resin groups. The results indicated no statistically significant difference in fluoride release after 3 and 6 days; however, the unmodified group exhibited slightly higher fluoride release than the group with 2% chitosan addition. Given the clinical relevance of fluoride release in preventing secondary caries, particularly in patients with varying levels of caries risk, this area remains underexplored. Further studies are needed to better understand how chitosan incorporation influences the fluoride-releasing potential of composite materials and to guide the selection of appropriate restorative materials for individual patient needs.

## 4. Materials and Methods

### 4.1. Focused Question

The systematic review followed the PICO framework [65] as follows: in cases of composite restorative materials (population), how does the addition of chitosan (investigated condition) affect their properties (outcome) in comparison to composite materials without modification? (comparison condition)?

### 4.2. Protocol

**Figure 2:**
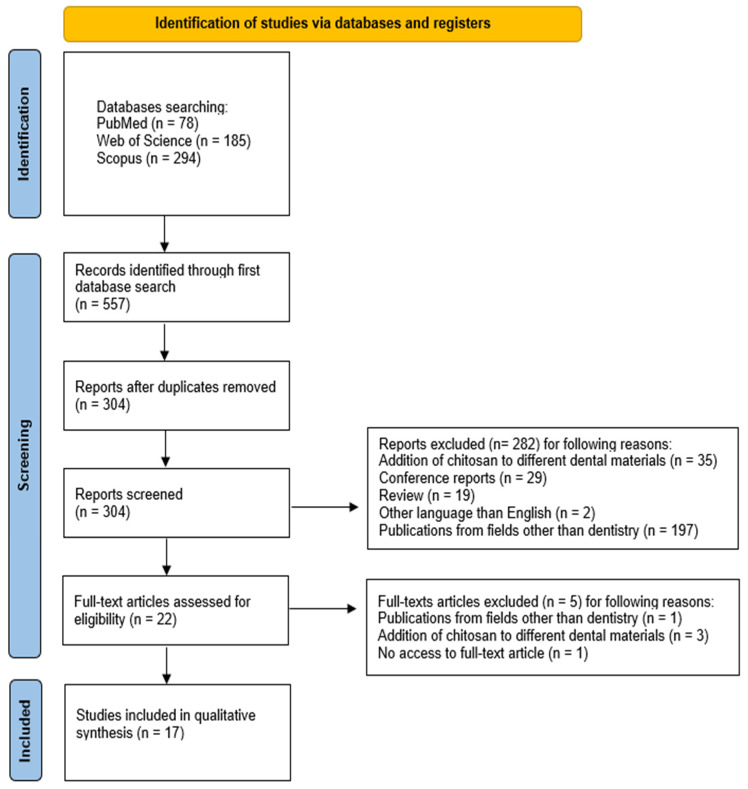
PRISMA flow diagram.

The selection process for articles included in the systematic review was carefully outlined following the PRISMA flow diagram [66] (see Figure 2). The systematic review was registered on the Open Science Framework under the following link: https://osf.io/bhxfv (accessed on 31 March 2025).

### 4.3. Eligibility Criteria

The researchers agreed to include only the articles that met the following criteria [67,68,69,70,71,72,73,74,75,76]:

- Addition of chitosan to the composite material;
- Research in the field of dentistry;
- In vitro studies;
- Studies in English;
- Full-text articles.

The exclusion criteria the reviewers agreed upon were as follows:

- Use of chitosan other than as an additive to the restorative material;
- Conference reports;
- Research in fields other than dentistry;
- Non-English papers
- Review articles;
- No full-text accessible;
- Duplicated publications.

No restrictions were applied with regard to the year of publication.

### 4.4. Information Sources, Search Strategy, and Study Selection

In March 2025, the PubMed, Scopus, and Web of Science (WoS) databases were searched to find articles meeting the specified inclusion criteria. To find articles focusing on composites with the addition of chitosan, the search was narrowed to titles and abstracts using a combination of keywords: resin AND composite AND chitosan. All searches conformed to the predefined eligibility criteria, and only articles with accessible full-text versions were taken into consideration.

### 4.5. Data Collection Process and Data Items

The articles that followed the inclusion criteria were carefully extracted by eight independent reviewers (P.P., M.M., J.K., A.K., W.Ś, J.K., S.K., W.D). The following data were used: first author, year of publication, study design, article title, addition of chitosan to the composite material, and its application in the field of dentistry. These essential data were entered into a standardized Excel file.

### 4.6. Risk of Bias and Quality Assessment

During the initial phase of study selection, each reviewer independently examined the titles and abstracts to mitigate potential reviewer bias. Cohen’s k test served as a tool to assess the extent of the agreement among reviewers. Any discrepancies regarding the inclusion or exclusion of an article in the review were addressed through discussion among the authors [77].

### 4.7. Quality Assessment

Two independent reviewers (W.D, J.M.) evaluated the procedural quality of each study included in the article. The assessment criteria were based on the presence of key information related to the association of Dental Resin Composites Modified with Chitosan. To evaluate the randomization, sample size calculation, control group, detailed description of the percentage of chitosan in the material, description of specimen manufacturing, application of standardized procedures (ISO), blinding, and number of research methods applied (microbiological/mechanical/ physicochemical), the studies were scored on a scale from 0 to 10 points, with a higher score indicating higher study quality. The risk of bias was assessed as follows: 0–4 points denoted a high risk, 5–7 points denoted a moderate risk, and 8–10 points indicated a low risk. Any discrepancies in scoring were resolved through discussion until a consensus was reached.

## 5. Conclusions

The aim of the study was to determine whether chitosan-modified resins may be incorporated in dental practices and how they modify features of dental restorations. Chitosan improves dental resin properties under specific conditions, such as proper concentration with lower concentrations (0.2–2%), providing optimal benefits without compromising mechanical integrity. The studies showed that 2% chitosan/fluoride particles improved antibacterial properties while maintaining mechanical strength. Conversely, higher concentrations (>2%) typically resulted in deteriorated mechanical properties. Moreover, the method of formulation proves crucial to effectiveness, with research demonstrating that glutaraldehyde crosslinking significantly enhances antimicrobial efficacy. Precise processing techniques are essential to achieve homogeneous chitosan distribution throughout the resin matrix, while the molecular weight of chitosan directly influences its reactivity with composite components. The primary benefit is antimicrobial activity, with six studies confirming the inhibition of cariogenic bacteria, including S. mutans, S. sanguinis, and L. acidophilus. Additionally, several studies reported improved fracture toughness and microhardness, potentially extending restoration longevity. In addition, no results presented a higher or lower capacity of fluoride release in chitosan-modified resins. However, limitations exist with higher chitosan concentrations, which decreased the flexural strength in three studies and increased microleakage in one study. Bond strength remained unaffected at low concentrations but decreased significantly at concentrations above 1%. In conclusion, chitosan incorporation is most beneficial at lower concentrations (0.2–2%), where it provides antimicrobial benefits without compromising the critical mechanical properties necessary for clinical performance.

This systematic review concludes that chitosan can be successfully incorporated into dental resins with clear evidence of technical workability across various formulations including powders, microparticles, and nanoparticles, offering promising benefits, particularly antimicrobial properties and increased fracture hardness, but recommends maintaining relatively low concentrations to preserve the critical mechanical properties necessary for the clinical performance and longevity of dental restorations.

## Figures and Tables

**Table 1:** General characteristics of studies.

Study	Aim of the study	Materials and Methods	Results	Conclusions
Lai et al. [44]	Assessment of the antibacterial properties, fluoride release, and recharge of fissure sealant containing C/F microparticles.	C/F microparticles were incorporated into the Bis-GMA matrix. The experimental group consisted of samples with the addition of 0%, 2%, and 4% C/F, with ClinproTM fissure sealant as a control group. Antibacterial activity was detected by Alamar Blue assay and colony-forming units (CFU). Biocompatibility was evaluated using WST-1 and LDH tests. ISO standards were applied to measure curing depth, flowability, tensile and flexural strength, while microhardness was determined by the Vickers test. Fluoride release and recharge were analyzed using ionic chromatography.	2% and 4% C/F demonstrated antibacterial properties, reducing CFU ratios to 10% and 25%, respectively (*p* < 0.01). However, 4% C/F showed cytotoxicity (*p* < 0.001). 2% C/F outperformed ClinproTM in curing depth (*p* < 0.001), microhardness, and tensile strength (*p* < 0.01), with comparable fluoride release and recharge ability (*p* = 0.67).	Resin with the addition of 2% C/F was indicated as an antibacterial sealant with satisfactory mechanical strength, present fluoride release, and the ability to recharge.
Ali et al. [45]	Exploration of antibacterial activity and hydrolytic stability of resin dental composite restorative materials (RDCRM) containing CS.	The antibacterial activity of microhybrid and flowable RDCRM with 0, 0.25, 0.5, and 1% wt/wt of CS against *L. casei* bacteria was evaluated via agar diffusion test and direct contact methods. Hydrolytic stability was assessed using gravimetric analysis.	The control and experimental flowable and microhybrid RDCRM showed no growth inhibition zone in the lawn growth of *L. casei*. The direct contact test revealed comparable CFU count of *L. casei* among the experimental and control RDCRM. Water sorption and solubility values for all RDCRM formulations remained within ISO standards, with no statistically significant differences between experimental groups.	The addition of one percent of CS in RDCRM had no antibacterial activity against *L. casei*. The hydrolytic stability of RDCRM containing CS was within the acceptable level.
Tanaka et al. [40]	Development of experimental composite resins with CS or CS loaded with DCPA possessing antimicrobial potential while preserving their mechanical properties and biocompatibility.	CS and CS/DCPA particles were synthesized using the electrospray method. The experimental biomaterials were formulated by adding particles (0, 0.5, or 1.0 wt%) into a resin matrix. Conversion degree and mechanical properties were assessed after 1 and 90 days of water aging post-photoactivation. Dental pulp fibroblasts in conditioned medium were used to evaluate cytotoxicity and genotoxicity. *S. mutans* antimicrobial activity was determined via crystal violet biofilm assay.	Experimental composites showed no cytotoxicity or genotoxicity. All chitosan-containing formulations reduced biofilm by 20% (*p* < 0.001) compared to the control group. With no detectable CS release, antimicrobial effects likely resulted from direct bacterial contact with exposed surface particles. The addition of the CS and CS/DCPA submicrometer particles to restorative materials maintained the degree of conversion, flexural strength, elastic modulus, and fracture toughness comparable to the control group after 90 days of aging in water.	The addition of CS or CS/DCPA particles provided antimicrobial properties to the composites without compromising mechanical properties or biocompatibility.
Ali et al. [18]	Evaluation of the antibacterial activity and the hardness of microhybrid and flowable resin-based composites (RBC) modified with CS.	RBC (flowable and microhybrid composites) were doped by CS in the form of flakes or powder. Number of 40 specimens were subjected to testing (20 flow, 20 microhybrid, both with 0, 0.25, 0.5, and 1.0% of CS addition)	No growth inhibition zones were observed for actinomyces, with comparable CFU counts between experimental and control groups. CS-modified composites demonstrated higher VH (Vickers Hardness), indicating increased surface resistance.	The addition of CS improved mechanical capabilities without improving antibacterial activity.
Kim and Shin [46]	Evaluation of antibacterial effect and mechanical properties of RBC incorporating CS.	*S. mutans* CS powder was inoculated in Brain Heart Infusion (BHI) solution, centrifuged (12 h), and absorbance measured for antibacterial assessment. Mechanical properties were evaluated via Vickers hardness and 3-point bending tests after 1 and 3 weeks of storage, respectively.	CS powder inhibited *S. mutans* growth, reducing CFU. Mechanical properties showed no significant differences, though one resin exhibited slightly decreased flexural strength and maximum load.	CS addition to restorative materials, provides antibacterial benefits without compromising mechanical properties.
Kikuchi et al. [47]	Evaluation of the influence of CS particles loaded with DCPA on the mechanical properties, degree of conversion, and mechanical properties of resin-based composites.	CS particles were added to experimental resins and separately characterized by Minimum inhibitory concentration (MIC) against *S. mutans*.	Composites demonstrated significantly higher biofilm inhibition. Particle-containing composites showed no significant decrease in flexural strength.	CS enhanced antibacterial activity without affecting mechanical properties compared to the control group
Fadhila et al. [19]	The impact of CS and CA on composite resin’s mechanical properties, toxicity, and antibacterial activity was investigated.	9 resin samples with CA+ CS were tested for: dissolution/water absorption in saliva; microleakage via methylene blue penetration; compressive strength, SBS, and tensile strength; hardness via VH; thermal properties by oven incubation. Toxicity assessed by shrimp larvae viability; antibacterial activity against S. mutans.	CS+CA inhibits *S. mutans* growth and is non-toxic at low doses. CS+CA addition reduces SBS, hardness, and water absorption; increases solubility, compressive, and tensile strength. No significant difference in microleakage or shrinkage compared to the control.	CS and CA addition enhances antibacterial and mechanical properties without inducing toxicity in the filling material.
Masoumi et al. [48]	Assessment of the effect of adding CS to composite resin on material properties.	Composite resin without CS and with 1% CS in the form of discs was obtained. Antibacterial effect—samples were incubated in *S. mutans* suspension. Biofilm and growth inhibition zones were assessed; resin conversion was measured using a spectrophotometer. Water solubility and sorption assessment of sample mass before and after immersion in water; SBS assessment - resin cylinders were glued to maxillary molars, and the wire and loop method was used.	The addition of CS increased the inhibition of biofilm development on the resin surface and significantly increased the water sorption of the composite. No significant effects were observed on composite conversion degree or SBS.	Incorporating CS into composites confers antibacterial properties without affecting mechanical properties.
Chander and Venkatraman [49]	Investigation of the influence of CS addition on the mechanical properties of denture base resin.	Four composite material samples were prepared: without CS and three samples with CS (5, 10, 15%). They were tested for: flexural and fracture strength; impact strength; surface roughness.	After the addition of CS to the resin, flexural strength, fracture strength, and impact strength increased. When the resin reached 15% of CS, the surface roughness decreased.	The addition of CS to dental resin can improve the mechanical parameters and increase its smoothness.
Harmaji et al. [50]	Determination of how varying CS concentrations affect the mechanical properties of alumina–zirconia–carbonate apatite nanoparticle composites.	Alumina-zirconia synthesized by sol-gel (900°C calcination); carbonate apatite from calcium nitrate, ammonium hydrogen phosphate, sodium hydrogen carbonate (700 °C calcination); CS solutions (2–6% in acetic acid); dental composite: dimethacrylate matrix and fillers (alumina-zirconia/carbonate apatite 50:50) at 70:30 ratio; light-cured samples tested for hardness, morphology, and composition.	When CS concentration was higher, particle sizes increased. The addition of 2% CS provided the most uniform particle size distribution; higher concentrations caused aggregation. Hardness decreased with increasing CS addition. Only 2% chitosan met dental material hardness standards (30–90 VHN).	The incorporation of CS has been demonstrated to exert an influence on the mechanical properties of the composite. A lower concentration of CS (2%) was found to be optimal for composite manufacturing, producing smaller nanoparticle sizes, improved distribution of the particles, and higher hardness values.
Halkai et al. [51]	Evaluation of whether the addition of 0.2% CSN (chitosan nanoparticles) to either universal or bulk-fill composites would improve fracture resistance across various cavity geometries in maxillary premolars.	Number of 130 maxillary premolars were divided as follows: control (*n* = 10), class I (*n* = 40), class II MO (*n* = 40), class II MOD (*n* = 40). Each cavity group is subdivided: A: Neo spectra ST-Universal composite, B: Tetric N-Ceram Bulk-fill composite, C: NST+0.2% CSN, D: TNC+0.2% CSN. CSN prepared in distilled water/acetic acid. All cavities received G-Premio Bond adhesive.	MOD cavities exhibited the highest fracture resistance, followed by MO cavities, then class I. TNC bulk-fill with CSN consistently demonstrated the highest fracture resistance across all cavity designs.	The addition of CSN improved fracture resistance in all cavity designs. TNC bulk-fill with 0.25% CSN showed the best overall performance.
Deb et al. [52]	Microleakage evaluation of microhybrid composite and 0.2% CS-doped composite in Class V cavities.	60 maxillary premolars with Class V cavities were divided into two groups (*n* = 30): microhybrid or CS-incorporated composite. Each group contained two subgroups (*n* = 15): immediate testing or testing after three months in artificial saliva. Microleakage was evaluated via dye extraction and spectrophotometry.	After immediate restoration, no significant microleakage difference existed between CS-modified and unmodified composites. Following 3 months in artificial saliva, microleakage significantly increased in unmodified composites while remaining stable in CS-modified composites.	CS-incorporated composite demonstrated superior stability, enhanced mechanical properties, and antibacterial advantages versus the unmodified microhybrid composite.
Deb et. Al. [53]	In vitro microleakage evaluation of 0.2% CS-enriched composite resin compared to microhybrid composite resin using a two-etching system.	Class V cavities in 60 human teeth were divided into 4 groups: two with the total-etch system and two with the self-etch system. Each group received either traditional or experimental material. Microleakage was measured using dye and spectrophotometry.	A significant difference favoring the CS-enriched composite was observed with the total-etch method. No significant difference was found between total-etch and self-etching systems.	The addition of CS did not interfere with the bonding of the composite resin to dentin.
Stenhagen et al. [54]	Synthesis of methacrylated chitosan (CH-MA) and its addition to dental composite. Assessment of mechanical properties and biofilm inhibition of the biomaterial.	The experimental composite was prepared by partial filler replacement with CH-MA. Resulting disks were used for *S. mutans* biofilm testing, Kaiser test analysis, and mechanical property evaluation.	CS was observed to have a significant impact on the reduction of *S. mutans* biofilm formation and composite hardness, as compared with the control group.	Incorporation of CH-MA into the experimental composite reduced *S. mutans* biofilm formation. An increased amount of CH-MA negatively affected the mechanical properties of the material.
Niu et al. [55]	Investigation of the mechanical and chemical properties of a bioactive composite resin that contains phosphorylated CS and amorphous calcium phosphate (Pchi/ACP).	Pchi was synthesized by the Nashi method. Composite was prepared by incorporating freeze-dried Pchi/ACP. The material was employed in the fabrication of disks for the purpose of conducting a series of experiments. The experiments conducted in this study encompassed the assessment of calcium ion release, hardness, wetting angle, modulus of elasticity, flexibility, and remineralization properties. Moreover, scanning electron microscopy (SEM) images were obtained to enable subsequent analysis.	Increasing Pchi/ACP content produced smoother surfaces, reduced wetting angles, decreased hardness and elasticity, while enhancing calcium ion release and dentin remineralization.	The mechanical and chemical properties of the composite deteriorate with the addition of Pchi/ACP.
Halkai et al. [56]	Bond strength evaluation of a composite containing different concentrations of chitosan nanoparticles (CSN).	Class II cavities were made in 70 molars and then filled with composite containing 0.25% and 2% CSN. Push-out bond strength was also tested.	The highest bond strength was observed when using the composite with 0.25% CSN content. However, it was not statistically different from the control group.	The investigation revealed that the incorporation of 0.25% CSN into a composite resin did not exert any significant influence on the push-out bond strength of class II restorations, as compared to the control material.
Farzanegan et al. [57]	Assessment of how varying concentrations of CSN and TiO_2_ NPs influence the shear bond strength (SBS) of an orthodontic adhesive.	72 extracted human premolars embedded in an acrylic resin and randomly allocated into four groups (*n* = 18). Gr. 1 (control): Brackets bonded with Transbond XT orthodontic adhesive Gr. 2: Bonding with Transbond XT containing 0.5% CSN and 0.5% TiO_2_ (anatase) NPs; Gr 3.: Bonding with Transbond XT containing 1% CSN and 1% TiO2 (anatase) NPs; Gr. 4: Bonding with Transbond XT containing 1.5% CSN and 1.5% TiO_2_ (anatase) NPs. SBS and ARI were evaluated using a universal testing machine.	No statistically significant differences in SBS were found among groups 1, 2, and 3, while group 4 showed significantly decreased SBS. Increasing concentration to 1% (both chitosan and TiO₂) did not significantly affect SBS; however, at 1.5% concentration, SBS was lower compared to other groups. No significant differences in ARI scores were observed between groups.	The orthodontic composite containing 1% CSN and 1% TiO2 NPs has adequate SBS for use in the clinical setting.

**Table 2:** Detailed characteristics of studies.

Authors	Chitosan Concentration	Tested Properties	Effect on Bacterial Biofilm	Influence on Mechanical Properties	Influence on Shear Bond Strength	Fluoride Release Capacity
Lai et al. [44]	0, 2, 4% C/F microparticles addition into the resin	Particle size, antibacterial properties, cell survival, and mechanical properties	Bis-GMA reduces metabolic activity and biofilm biomass.	The incorporation of 2% of C/F particles has resulted in an increase in the curing depth, microhardness, and tensile strength of the material. There was a decrease in flowability, whilst flexural strength was observed to remain unchanged. Mechanical strength 73.18 MPa	No data	Comparable to sole sealant
Ali et al. [45]	RDCRM containing 0, 0.25, 0.5, and 1 wt% of chitosan	Antibacterial properties and water sorption	No antibacterial effect against *L. casei* bacteria.	No data	No data	No data
Tanaka et al. [40]	0.5 and 1 wt% addition of chitosan and CS/DCPA particles	Cytotoxicity and genotoxicity, chitosan release, antimicrobial activity, mechanical properties, degree of conversion, elemental analysis	Both the CS and CS/DCPA containing composites demonstrated antimicrobial properties against *S. mutans*.	Following a 90-day hydration period, the mechanical properties of all composites were found to be equivalent, indicating that the presence of CS or CS/DCPA submicrometer fillers did not exert a detrimental influence on the composites’ mechanical properties. Flexural strength 85 MPa	No data	No data
Ali et al. [18]	0, 0.25, 0.5, and 1%	Antibacterial properties and hardness	No antibacterial activity has been presented. The control and experimental groups were comparable.	VH was significantly higher in the group with flowable RBC modified with 1% chitosan. Group consisting of microhybrid RBC modified with 0, 5% CS demonstrated significantly higher VH. Vickers hardness: Control (MH0) = 49 VHN MH0.25 = 48 VHN MH0.5 = 50 VHN MH1.0 = 52 VHN Control (FA0) = 82 VHN FA0.25 = 77 VHN FA0.5 = 83 VHN FA1.0 = 75 VHN	No data	No data
Kim and Shin [46]	Three CS powders with different molecular weights: low, medium, and high (75–85% deacylated powder)	Antibacterial properties, inhibitory effect on *S. mutans*, bending test, hardness, flexural strength, flexural modulus	The effect of CS on *S. mutans*: absorbance was significantly higher. CFUs in chitosan-modified groups were significantly lower.	There was no significant difference in VH and flexural modulus. Maximum load and flexural strength were significantly lower in CS modified resins. Flexural strength: Control = 108.3 MPa L = 86.28 MPa M = 64.02 MPa H = 89.36 MPa Vickers hardness: Control = 32.63 VHN L = 32.04 VHN M = 29.63 VHN H = 29.72 VHN	No data	No data
Kikuchi et al. [47]	CS/DCPA was added in 0, 5% concentration to the composite resin. Crosslinking times were 0, 8, and 16 h.	Mechanical properties, degree of conversion, and antibacterial properties	Composite resins modified with CS/DCPA crosslinked for 16 h showed the highest percentage of dead bacteria. The amount of dead cells was higher in all modified resins in comparison with the control group.	Flexural strength of modified resins was lower for the group crosslinked for 8 h. The storage of the material in water has been shown to reduce the flexural strength of the material with non-crosslinked particles in comparison with storage in a dry state after 24 h and seven days. The storage in acid reduced the flexural strength in the material with particles crosslinked for 8 h after 7 days. Regarding elastic modulus, it was determined that neither the degree of crosslinking of the particles nor the storage medium had a significant impact on the flexural strength of the investigated materials. Flexural strength: Control = 81 MPa 0 h = 74 MPa 8 h = 83 MPa 16 h = 77 MPa Elastic modulus: Control = 3.2 GPa 0 h = 3.1 GPa 8 h = 2.8 GPa 16 h = 3.4 GPa	No data	No data
Fadhila et al. [19]	3, 5, 7% of CS addition in the matrix containing CA	Solubility, water absorption, microleakage, compressive strength, shear bond strength, tensile strength, hardness, toxicity, antibacterial properties, thermal expansion/shrinkage	Mean zone of inhibition of *S. mutans* growth (mm): Cellulose (12,80); CS (8,70); Cellulose + CS (7,57); negative control (0,00); positive control (20,10)	Reduction in SBS, hardness, and water absorption; Increase in solubility, compressive strength, and tensile strength. No change: microleakage, shrinkage Vickers hardness: Control = 33.9 VHN Samples with chitosan: 0.4–1.17 VHN	No significant differences between the control and experimental groups were observed	No data
Masoumi et al. [48]	1% of CS addition	Antibacterial properties, water absorption, solubility, and SBS	CFU: 1. *S. mutans*—control: 13.800; composite with CS 5.971 2. *S. sanguinis*—control: 19.400; composite with CS 7.014 3. *L. acidophilus*—control: 16.885; composite with CS: 7.728	No data	Micro SBS values ( MPa): 1. Control: 6.122 2. Pretreatment: 5.382 3. Adhesive + CS: 6.304 4. Composite + CS: 5.739	No data
Chander and Venkatraman [49]	5, 10, 15%	Flexural strength, fracture strength, impact strength, surface roughness	No data	Improvement of flexural strength, fracture strength, and impact strength Flexural strength 0% = 67.890 MPa 5% = 73.019 MPa 10% = 71.903 MPa 15% = 69.253 MPa	No data	No data
Harmaji et al. [50]	2, 4, and 6% addition of CS	Hardness, particle size, morphology, and phase composition	No data	Lower chitosan concentration improves the mechanical properties of composites: 2%—51.3 VHN, 4%—28.24 VHN, 6%—25.48 VHN	No data	No data
Halkai et al. [51]	0.2% addition of CSN	Fracture resistance	No data	No data	No data	No data
Deb et al. [52]	0.2% addition of CS	Microleakage	No data	The composite demonstrated a statistically significant increase in microleakage, from 0.0105 nm to 0.0158 nm over a duration of three months. In contrast, CS + composite demonstrated a comparatively minor increase from 0.0096 nm to 0.0117 nm, exhibiting no statistically significant difference	No data	No data
Deb et al. [53]	0.2% addition of CS	Microleakage	No data	No data	No data	No data
Stenhagen et al. [54]	5, 10, 20% addition of CH-MA	Antibacterial effect, flexural strength, hardness, amount of free amino groups, SBS	The addition of 10–20% CH-MA to composites resulted in a reduction in *S. mutans* biofilm formation at pH 5.9 after 24 h and two weeks (in comparison with control groups), with no effect observed at pH 7. However, 20% CH-MA in adhesives led to a decrease in biofilm at pH 7 after 24 h.	Flexural strength Control = 131.0 MPa 5% = 87.0 MPa 10% = 72.0 MPa 20% = 65.8 MPa Hardness Control = 56.8 (3.0) HV 5% = 43.7 (1.2) HV 10% = 39.5 (1.2) HV 20% = 25.9 (1.1) HV	Control = 24.0 MPa 10% = 22.8 Mpa 20% = 22.4 MPa	No data
Niu et al. [55]	15, 25, 35% addition of Pchi/ACP	Morphology, contact angle, flexural strength, elastic modulus, hardness, calcium ion release, remineralization	No data	Flexural strength, elastic modulus, and hardness decreased with the addition of CS No numeric data	No data	No data
Halkai et al. [56]	0,25 or 2%	Push-out bond strength	No data	No data	Mean value Control = 568.8 (139.80)N 0,25% = 623.2 (60.52)N 2% = 475.4 (33.85)N	No data
Farzanegan et al. [57]	0.5, 1.0, and 1.5% of CSN and TiO_2_ NPs	SBS, ARI	No data	ARI scores were not statistically significant between groups	The addition of 1.5% CSN and 1.5% TiO_2_ NPs resulted in a decrease in SBS compared to the other three groups	No data

**Table 3:** Quality assessment of reviewed articles.

Authors/ Criteria	Randomization	Sample Size Calculation	Control Group	Detailed Description of the Percentage of Chitosan in the Material	Description of Specimen Manufacturing	Application of Standardized Procedures (ISO)	Blinding	Number of Research Methods Applied (Microbiological/Mechanical/ Physicochemical): 1 method— 1 point 2 methods— 2 points 3 methods— 3 points	Total Points	Risk of Bias
Lai et al. [44]	0	0	1	1	1	1	0	3	7	moderate
Ali et al. [45]	0	0	1	1	1	1	0	2	6	moderate
Tanaka et al. [40]	0	0	1	1	1	1	0	3	7	moderate
Ali et al. [18]	0	0	1	1	1	1	0	2	6	moderate
Kim and Shin [46]	0	0	1	1	1	1	0	2	6	moderate
Kukuchi et al. [47]	0	0	1	1	1	0	0	3	6	moderate
Fadhila et al. [19]	1	0	1	1	1	0	0	3	7	moderate
Masoumi et al. [48]	0	1	1	1	1	1	0	3	8	low
Chander and Venkatraman [49]	0	0	1	1	1	1	0	2	6	moderate
Harmaji et al. [50]	0	0	0	1	1	0	0	2	4	high
Halkai et al. [51]	0	0	1	1	1	0	0	1	4	high
Deb et al. [52]	0	0	1	1	1	0	0	1	4	high
Deb et al. [53]	1	0	1	1	1	0	0	1	5	moderate
Stenhagen et al. [54]	0	0	1	1	1	1	0	3	7	moderate
Niu et al. [55]	0	0	1	1	1	0	0	2	5	moderate
Halkai et al. [56]	0	0	1	1	1	0	0	1	4	high
Farzanegan et al. [57]	1	1	1	1	1	0	1	1	7	moderate

## Data Availability

Not applicable.
